# Homeology of sex chromosomes in Amazonian *Harttia* armored catfishes supports the X-fission hypothesis for the X_1_X_2_Y sex chromosome system origin

**DOI:** 10.1038/s41598-023-42617-w

**Published:** 2023-09-21

**Authors:** Francisco de Menezes Cavalcante Sassi, Alexandr Sember, Geize Aparecida Deon, Thomas Liehr, Niklas Padutsch, Osvaldo Takeshi Oyakawa, Marcelo Ricardo Vicari, Luiz Antonio Carlos Bertollo, Orlando Moreira-Filho, Marcelo de Bello Cioffi

**Affiliations:** 1 Laboratório de Citogenética de Peixes, Departamento de Genética e Evolução, Universidade Federal de São Carlos, São Carlos, SP 13565-905 Brazil; 2 Laboratory of Fish Genetics, Institute of Animal Physiology and Genetics, Czech Academy of Sciences, Rumburská, 89, Liběchov, Czech Republic; 3 Institut für Humangenetik, Universitätsklinikum Jena, 07747 Jena, Germany; 4 Museu de Zoologia, Universidade de São Paulo, São Paulo, SP 04263-000 Brazil; 5 Departamento de Biologia Estrutural, Molecular e Genética, Universidade Estadual de Ponta Grossa, Ponta Grossa, PR Brazil

**Keywords:** Evolutionary biology, Evolutionary genetics

## Abstract

The Neotropical monophyletic catfish genus *Harttia* represents an excellent model to study karyotype and sex chromosome evolution in teleosts. Its species split into three phylogenetic clades distributed along the Brazilian territory and they differ widely in karyotype traits, including the presence of standard or multiple sex chromosome systems in some members. Here, we investigate the chromosomal rearrangements and associated synteny blocks involved in the origin of a multiple X_1_X_2_Y sex chromosome system present in three out of six sampled Amazonian-clade species. Using 5S and 18S ribosomal DNA fluorescence in situ hybridization and whole chromosome painting with probes corresponding to X_1_ and X_2_ chromosomes of X_1_X_2_Y system from *H. punctata*, we confirm previous assumptions that X_1_X_2_Y sex chromosome systems of *H. punctata*, *H. duriventris* and *H. villasboas* represent the same linkage groups which also form the putative XY sex chromosomes of *H. rondoni*. The shared homeology between X_1_X_2_Y sex chromosomes suggests they might have originated once in the common ancestor of these closely related species. A joint arrangement of mapped *H. punctata* X_1_ and X_2_ sex chromosomes in early diverging species of different *Harttia* clades suggests that the X_1_X_2_Y sex chromosome system may have formed through an X chromosome fission rather than previously proposed Y-autosome fusion.

## Introduction

Teleost fishes display an astounding variety of sex determination systems, involving genetic and environmental mechanisms or a combination thereof^1–3^. These mechanisms may largely differ among fish lineages and even among populations of the same species^4–7^. Particularly genetic sex determination is in fishes mainly governed by one of the nine presently known sex chromosome systems which evolved independently multiple times in different lineages^6–9^ and carry different sex-determining genes^2,10^. The majority of these systems show little genetic differentiation^8,11,12^ and are therefore prone to frequent sex chromosome turnovers^7,13–15^. These properties make teleost sex chromosomes a well-suited model for studying early phases of sex chromosome differentiation^16,17^ and causes and consequences of sex chromosome turnovers (whereby the newly evolved system replaces the former one)^15,18,19^, and the bearing of sex chromosome evolution to the establishment of reproductive barriers between incipient species^20–22^.

The study of sex chromosomes has undergone a remarkable transformation in recent years as genome sequencing, assembly and scaffolding techniques rapidly improved. Despite these advances, several unique biological features of sex chromosomes are still hardly tractable by computational tools, or their analysis requires multiple integrated methodologies and/or large number of individuals to be analyzed^18,23^. Cytogenetics and particularly the use of whole chromosome painting (WCP) probes enables comparative study among multiple (closely) related species, and it can be narrowed down specifically to linkage group(s) representing sex chromosomes^8,24^. This approach enables to determine whether sex chromosomes originated independently from different linkage groups or are formed by the same synteny blocks. The latter situation points either on a single shared origin of sex chromosomes or repeated and independent co-option of the same synteny blocks for the sex-determining role^8–10^. The use of cytogenetics may also avoid misinterpretations related to accidental involvement of sex-reversed individuals or the intra-specific variability in the sex-determining systems^8,24^.

The Neotropical armored catfish genus *Harttia* (Siluriformes, Loricariidae, Loricariinae) presently harbors 28 valid species^25–27^ together with three *Harttia* spp. determined based on cytogenetic features but waiting for a proper taxonomic description^28^. After *Rineloricaria* (reviewed in^29^), *Harttia* displays the second-largest variation in diploid chromosome number (2n) among Loricariidae fishes, ranging from 2n = 52♀/53♂ in *H. carvalhoi*^30^, to 2n = 62♀♂ in *H. absaberi*^31^ and *Harttia* sp. 2^32^. Furthermore, the following three male-heterogametic sex chromosome systems have been identified in a subset of surveyed species: XX/XY_1_Y_2_ in *H. carvalhoi*, *H. intermontana*, and *Harttia* sp. 1^30,32,33^; X_1_X_1_X_2_X_2_/X_1_X_2_Y in *H. duriventris*, *H. punctata*, and *H. villasboas*^34,35^, and a putative XX/XY in *H. rondoni*^35^. Together with African *Nothobranchius* killifishes^36^, *Harttia* represents a genus with the highest incidence of multiple sex chromosomes among teleosts to date^8,32^. It is therefore highly informative lineage for investigating underlying evolutionary forces that drive transitions from standard sex chromosomes (or other forms of sex determination) to multiple sex chromosome systems.

The most updated phylogeny of Loricariinae^37^, though not including all valid species, recognized the monophyly of *Harttia*, with the occurrence of three distinct evolutionary lineages: Clade I is composed of the species from Guyanese shield, Clade II includes the species from the Amazonian and Tocantins-Araguaia river basins, and Clade III harbors the species from the southern/southeastern Brazil. When complemented with a species set from a former phylogenetic study^38^ it is clear that *Harttia* species with known sex chromosomes, though nested within species lacking them based solely on a cytogenetic evidence, are grouped according to the type of their sex chromosome systems: those with an X_1_X_1_X_2_X_2_/X_1_X_2_Y system or tentative XX/XY sex chromosomes are placed in the clade II, and those with an XX/XY_1_Y_2_ system in the clade III^28,32^.

In our former studies, we demonstrated by WCP probes used in cross-species experiments (Zoo-FISH) that X_1_X_2_Y and XY_1_Y_2_ sex chromosome systems represent different linkage groups and therefore evolved independently^39–41^. While we also revealed by the same method the full or partial homeology between XY_1_Y_2_ systems among the three *Harttia* spp.^39,41^, similar information is lacking for XY and X_1_X_2_Y systems as yet. Indirect evidence based on ribosomal DNA (rDNA) physical mapping and comparative genomic hybridization (CGH) suggested that these systems might be potentially homeologous^35^. In this study, we aim to investigate the mechanism(s) of origin and the relationships between the sex chromosome systems in *Harttia* species belonging to the Amazonian clades I and II where three members are known to carry an X_1_X_2_Y sex chromosome system and a single species possesses putative XY sex chromosomes. We therefore probed altogether six related *Harttia* species with WCP probes derived from the X_1_ and X_2_ sex chromosomes of the X_1_X_2_Y system in *H. punctata* thus complementing our former study^40^. The analysis in the present study was complemented by mapping of rDNA clusters as these usually locate on *Harttia* sex chromosomes^34,35,42^. Our data show homeology between X_1_X_2_Y sex chromosome systems and also the putative XY sex chromosome system of *H. rondoni*. Among the two formerly proposed hypotheses on X_1_X_2_Y sex chromosome origin i.e. the Y-autosome fusion^34,40^ and X fission^35^, our results support the latter scenario.

## Results

**Figure 1:**
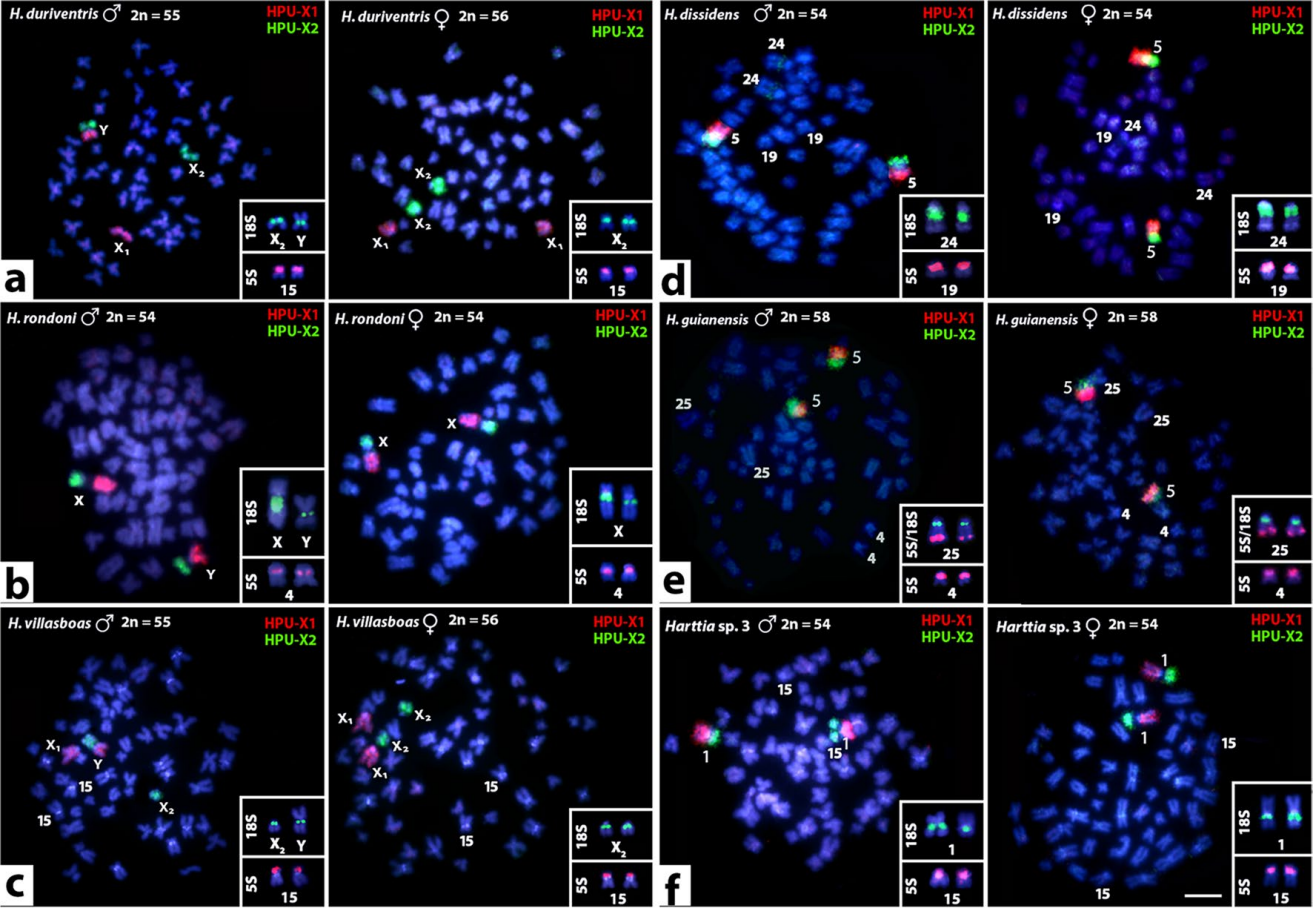
Zoo-FISH with HPU-X_1_ and HPU-X_2_ painting probes in male (first and third column) and female (second and fourth column) mitotic metaphases of *Harttia duriventris* (**a**), *H. dissidens* (**d**), *H. rondoni* (**b**), *H. guianensis* (**e**) *H. villasboas* (**c**), and *Harttia* sp. 3 (**f**). Chromosomes bearing 5S (red) and 18S (green) rDNA clusters as revealed after reprobing in the second FISH round are highlighted in boxes. The assignment of signals to specific chromosome pairs was performed based on data in our previous studies^28,35^. Full metaphase images are provided in Supplementary Fig. 1. Chromosomes were counterstained with DAPI (blue). Bar 10 µm.

Cross-hybridization with HPU-X_1_ and HPU-X_2_ painting probes revealed full homeology between X_1_X_2_Y sex chromosome systems of *H. punctata* (analyzed by us formerly^40^), *H. duriventris* and *H. villasboas* (Fig. 1a,c). In *H. duriventris* and *H. villasboas* the HPU-X_1_ probe entirely hybridized to X_2_ chromosome and conversely, HPU-X_2_ probe painted X_1_ chromosome when following the nomenclature by Sassi et al.^35^. As the location of sex-determining region has not been identified yet, the assignation of X_1_ (ancestral) and X_2_ (neo) sex chromosomes in different species was done arbitrarily in former studies^34,35^. Hence, to avoid confusion in designation of demonstrably the same synteny blocks, we unified the nomenclature of sex chromosomes according to X_1_X_2_Y system of *H. punctata*^34,40^.

The painting probes also labelled different portions of putative XY sex chromosome in *H. rondoni* (Fig. 1b) whose identity was confirmed by 18S rDNA mapping (see below). While the HPU-X_1_ probe painted the long (q) arms of these supposed X and Y chromosomes, the HPU-X_2_ probe painted short (p) arms of them both. Remarkably, a (peri)centric region of both chromosomes was left unstained by these probes. For the species without cytologically distinguishable sex chromosomes—*H. dissidens H. guianensis*, and *Harttia* sp. 3 (Fig. 1d–f) both painting probes, again, hybridized to a single metacentric chromosome pair. In *H. dissidens* and *H. guianensis* the hybridization pattern was the same as in *H. rondoni* (i.e. HPU-X_1_ covered q-arms while HPU-X_2_ stained p-arms of the fifth and fourth chromosome pair in *H. dissidens* and *H. guianensis*, respectively; compare Fig. 1d,e with Fig. 1b) but this time without the unstained region in (peri)centromeres. In *Harttia *sp. 3 (Fig. 1f) the painted chromosome corresponded to the 18S rDNA-bearing chromosome pair 1, with the HPU-X_1_ probe hybridizing on its p-arms and the HPU-X_2_ probe on its q-arms (i.e. the opposite scenario to the one found in *H. rondoni*, *H. dissidens* and *H. guianensis*). The (peri)centromeric region of this chromosome pair was left unstained by the painting probes.

18S rDNA signals were placed in the pericentromeric regions of X_2_ and Y chromosomes of *H. villasboas* and *H. duriventris* (Supplementary Fig. 1). These clusters also occupied (peri)centromeric positions in putative XY sex chromosomes in *H. rondoni.* Corroborating the previous study^35^, 18S rDNA cluster showed size heteromorphism with the X-linked site being notably extended compared to the Y-linked one. 18S rDNA probe further co-localized with the painting probes only in *Harttia *sp. 3 where it revealed a site on both homologs positioned on q-arms closely downstream of the centromere. Finally, in *H. dissidens* and *H. guianensis*, we observed a single pair of 18S rDNA-bearing chromosomes (pair 24 and 25, respectively, according to Sassi et al.^28^), with the centromere-proximal signal on the q-arms. In *H. guianensis*, the 18S rDNA-bearing chromosome pair also bore 5S rDNA cluster at the terminal portion of q-arms. Second pair of 5S rDNA signals in this species was located interstitially on the p-arms of metacentric chromosome pair 4. The same site was present also on chromosome pair 4 with the same morphology in *H. rondoni* where no additional 5S rDNA signals were detected. *H. villasboas*, *H. duriventris* and *Harttia* sp. 3 exhibited a single chromosome pair (no. 15) bearing 5S rDNA arrays on its p-arms. The same pattern but on chromosome pair 19 was found in *H. dissidens*.

**Figure 2:**
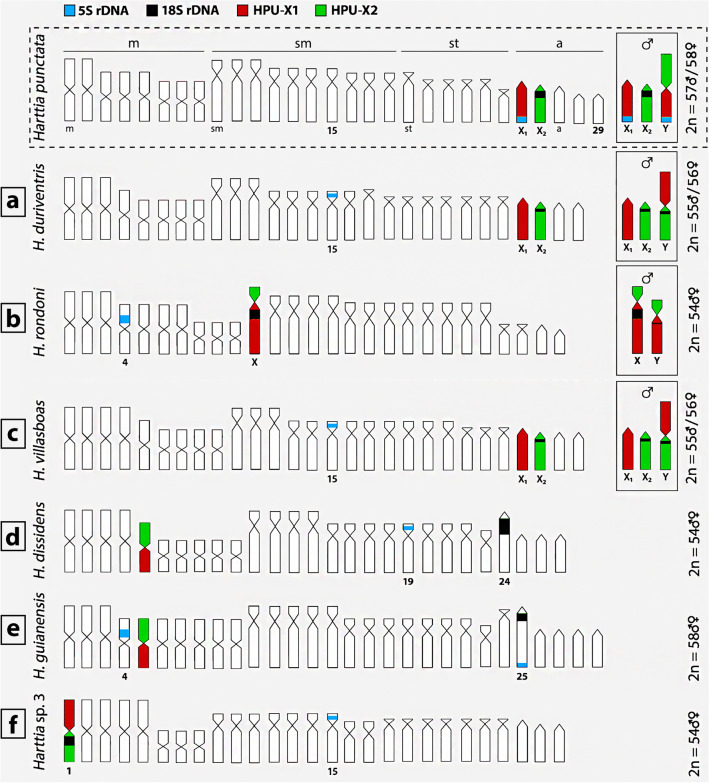
Schematic representation of hybridization results on chromosomes of studied *Harttia* species. For *Harttia punctata* the hybridization pattern was adopted from our previous study utilizing the same probes^40^. Letters correspond to those on Fig. 1. The assignment of signals to specific chromosome pairs, as well as the arrangement of chromosomes into a karyotype, was performed based on data in our previous studies^28,35^.

All hybridization patterns are summarized in an ideogram (Fig. 2). Full metaphase images with rDNA hybridization patterns are provided in the Supplementary Fig. 1.

## Discussion

We have shown herein by Zoo-FISH that *Harttia* species with X_1_X_2_Y sex chromosome system entirely share the synteny blocks by which these sex chromosomes are formed. Our findings also corroborate the existence of previously proposed^35^ XY sex chromosome system in *H. rondoni*, as also these chromosomes were stained by the same sex chromosome-derived painting probes.

Since the identification of the multiple XX/XY_1_Y_2_ sex chromosome system in *H. carvalhoi*^30^, *Harttia* catfish genus became an excellent model for studying sex chromosome evolution in Neotropical fishes, with three species having the X_1_X_2_Y sex chromosome system, other three the XY_1_Y_2_ one, and yet one another representative featuring tentative XY sex chromosomes. A series of cytogenetic studies relying mostly on repetitive DNA mapping, CGH and Zoo-FISH experiments provided already evidence that XY_1_Y_2_ systems found in *H. carvalhoi*, *H. intermontana* and *Harttia* sp. 1 are fully or partially homeologous among each other but are non-homologous to the X_1_X_2_Y system of *H. punctata*^28,32,34,35,39–42^. Homeology between X_1_X_2_Y sex chromosomes in *H. punctata*, *H. duriventris* and *H. villasboas*, and their close relationship to putative XY sex chromosomes in *H. rondoni* were previously proposed based on the shared presence of 18S rDNA clusters and CGH patterns^35^.

**Figure 3:**
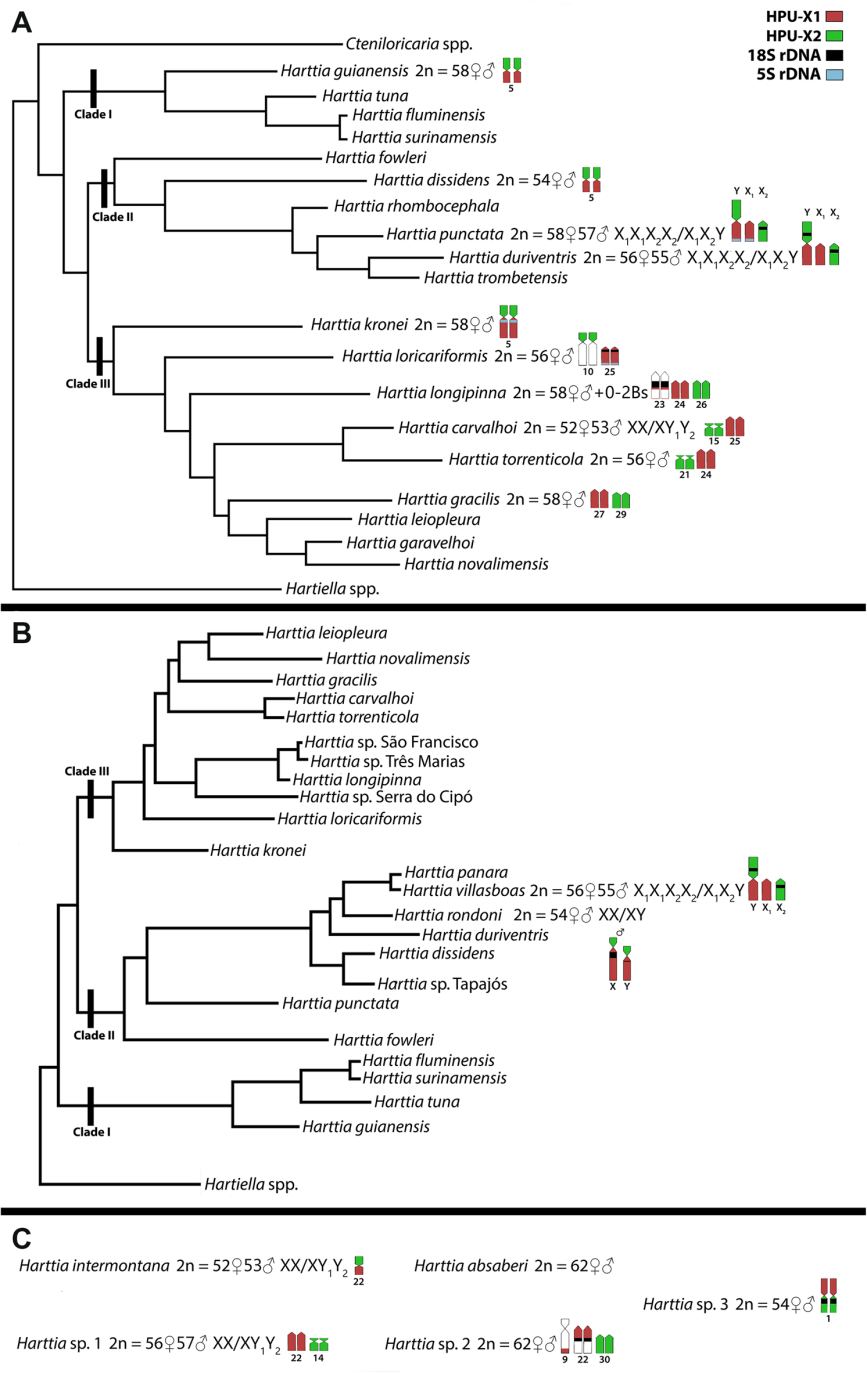
Phylogenetic relationships among Hartiini fishes based on morphological and molecular data along with anchored cytogenetic characteristics. Phylogeny follows^37^ (**a**), and^38^ (**b**), while (**c**) presents the chromosomal data from species that were not included in the respective phylogenetic reconstructions. Indicated cytogenetic characteristics: 2n; sex chromosome systems; partial ideograms represent the organization of mapped synteny blocks as revealed by the sex chromosome-derived painting probes HPU-X_1_ (red) and HPU-X_2_ (green); 5S rDNA (blue), and 18S rDNA (black) sites. The assignment of signals to specific chromosome pairs was performed based on data in our previous studies^28,32,35,40^. More specifically, the cytogenetic data synthesis has been undertaken as follows: *H. guianensis*, *H. dissidens* and *Harttia* sp. 3 (^28^this study); *H. punctata*, *H. kronei*, *H. loricariformis*, *H. longipinna*, *H. carvalhoi*, *H. torrenticola* and *H. gracilis*^40^; *H. duriventris*, *H. villasboas* and *H. rondoni* (^35^this study); *H. intermontana*, *Harttia* sp. 1 and *Harttia* sp. 2^32,40^; *H. absaberi*^31^.

Blanco et al.^34^ initially hypothesized that the X_1_X_2_Y sex chromosomes in *H. punctata* originated from a Robertsonian translocation between the two acrocentic chromosomes—an ancestral Y and an autosome. This rearrangement would be accompanied by the loss of 18S rDNA arrays from the emerging neo-Y chromosome. Nonetheless, the chromosome painting data from the work by Deon et al.^39^ and our present study, once anchored to the current phylogenetic analysis (^37^Fig. 3A, ^38^Fig. 3B), clearly show that the linkage groups representing X_1_ and X_2_ chromosomes were ancestrally forming arms of the same chromosome. Besides the early diverging *H. guianensis*, this pattern has been found also in other *Harttia* lineages (^39^Fig. 3). This means that more probably the X_1_X_2_Y system emerged after a centric fission in the ancestral X chromosome.

When a fission event creates an X_1_X_2_Y multiple sex chromosome system, a closely related species carrying the ancestral XY sex chromosomes is expected to have a lower 2n in the karyotype^43^. This is what can be inferred from the comparison of *H. rondoni* with 2n = 54 (XY/XX) and the species with multiple X_1_X_2_Y/♀X_1_X_1_X_2_X_2_ sex chromosomes: *H. punctata* with (2n = 57♂/58♀), *H. villasboas* and *H. duriventris* (2n = 55♂/56♀).

CGH analysis has formerly shown that a probable region of differentiation on the Y chromosome might be located proximally to the centromere^28^. Indeed, the centromeric regions have been widely shown to suppress recombination^44^ and therefore are thought to be suitable regions for establishment of a new sex-determining region^17^. It is further intriguing that *H. villasboas* and *H. duriventris* share the presence of centromere-proximal 18S rDNA site on their Y chromosomes and that both XY chromosomes in *H. rondoni* share a pericentromeric 18S rDNA cluster being consistently larger on the X chromosome (^35^this study). It is tempting to hypothesize that the ancestral situation would be close to the scenario found in *H. rondoni* and the X-linked amplified 18S rDNA region might cause instability around the centromere, leading eventually to the fission, while selection would counteract similar fission to happen on the Y chromosome, to preserve the linkage disequilibrium in/around the sex-determining region.

rDNA clusters have been abundantly shown to cause chromosomal instability due to heavy transcription and organization into long tandem arrays^45^. More specifically, the following conditions collectively provide ample opportunities for DNA damage to happen: (1) highly decondensed DNA, (2) exposure of non-templated DNA strand and its tendency to form various secondary structures and (3) increased probability of collision between transcription and replication machineries. Consequent DNA repair may accidentally lead to rearrangements^46–48^. In the frame of the X-fission scenario, loss of rDNA sequences is among the possible consequences of double-stranded breaks in these tandem repeats^49^. Moreover, fission itself may lead to a partial degradation of exposed chromosomal ends until new telomeres are being established^50^. rDNA dynamics in *Harttia* is further evidenced by a complete loss of rDNA sites on certain linkage groups and their emergence on another chromosome pairs (^40^this study—see Fig. 2). Notably, besides *Harttia* spp.^32,40^, rDNA sites operated as breakpoint regions independently in many fish groups^51–55^.

While *H. rondoni* has not been involved in the current phylogenetic analysis^37^, former work^38^ proposed the phylogenetic position of this species being nested within the species carrying X_1_X_2_Y sex chromosomes, which might either mean that (1) ancestral XY system has been preserved in *H. rondoni* while X_1_X_2_Y system evolved repeatedly in separate evolutionary events in the closely related species or (2) there was a single origin of X_1_X_2_Y system and X_1_ and X_2_ fused secondarily back again in *H. rondoni* creating a neo-XY system, or (3) the phylogenetic relationships of *H. rondoni* with closely related species are not interpreted correctly by Covain et al.^38^. Regarding the last point, although geographical distribution and morphological characters reinforce the Covain’s proposition^25^, further phylogenetic studies involving the species in question are necessary to untangle this issue. Noteworthily, if X_1_X_2_Y sex chromosomes evolved multiple times independently in this *Harttia* lineage, then the repeated fusion of ancestral Y with always the same autosome is rather improbable^9,56–58^, which, again, reinforces the X-fission hypothesis.

The origin of multiple X_1_X_2_Y sex chromosome system in the three *Harttia* species seems not to follow the common evolutionary pathway. A centric or tandem fusion of the original Y chromosome with an autosome has been proposed (and in several cases empirically confirmed) as an underlying mechanism leading to emergence of X_1_X_2_Y sex chromosomes in the remaining 62 teleosts cases reported to date^8,55,59^. It is also the commonest mechanism of multiple sex chromosome creation in other cold-blooded vertebrates^60,61^. Sex chromosome fissions are much less common and in teleosts they have been proposed thus far only in five cases (four times as Y-fission and once as W-fission; reviewed in^8^). Another tentative W-fission might have taken place in *Ancistrus clementinae*^62^. Finally, a Y-fission has been proposed also for the XY_1_Y_2_ system in *H. carvalhoi*^33^, however, a more recent study^32^ suggested X-autosome fusion to be responsible instead, which has been further reinforced by Zoo-FISH showing that a probable ancestral X chromosome fused with two different autosomes within the set of three XY_1_Y_2_-bearing *Harttia* species^39^.

Fissions are generally hard to track in the species' karyotypes as they generate less noticeable products compared to large chromosomes derived from fusions^50,63^. Hence, unfortunately, our understanding of the genomic properties of fission sites and the etiology of this rearrangement type are still rather limited, despite the steadily growing number of sequenced genomes in non-model organisms. Studies combining chromosomal painting with specific sex-chromosome probes and other cytogenetic markers, such as the distribution of repetitive DNAs, are useful to indicate major fission events, as already proven in fishes^64,65^, lizards^56,66^, and birds^67^. Such an approach is desirable as fissions have been associated with chromosomal evolution and speciation events in several metazoans^68–70^ and they have been also, for instance, associated with the evolution of the olfactory system in carnivores^71^.

While sex chromosome-autosome fusions have been much more explored regarding their possible effects on reproductive isolation, adaptation and radiation^8,20,57,60^ fissions might have a similar effect^72^. In the case of *Harttia* species, given that these fishes form rather small, fragmented populations with restricted/absent gene flow, multiple sex chromosomes might have emerged and get fixed rather under the major effect of genetic drift^73^ which highly likely applies also to several other Neotropical fishes with multiple sex chromosomes^55,65,74^. The possible contribution of natural selection in this process will require further studies particularly oriented towards a detailed characterization of genetic content of sex-determining regions and neighboring chromosomal areas.

## Conclusion

The chromosomal evolution in *Harttia* species has been shaped by numerous inter-chromosomal rearrangements giving rise to a complex karyotype variability, including the emergence of different male-heterogametic sex chromosome systems. In this study, we demonstrated that the X_1_X_2_Y and XY sex chromosomes, as well as some autosomes, share several homologies among *Harttia* species. This strengthens our previously proposed theory that the X_1_X_2_Y system emerged after a centric fission in the ancestral X chromosome and thus represents a derivation of the ancestral XY sex chromosome system. Although the amount of chromosomal data has significantly increased for *Harttia* species during the recent years, the genus still lacks a robust phylogenetic reconstruction that would include all recognized species along with the other emerging but yet undescribed species whose existence has been proposed by their distinct cytogenetic features^28^.

## Methods

### Sampling and chromosome preparation

**Figure 4:**
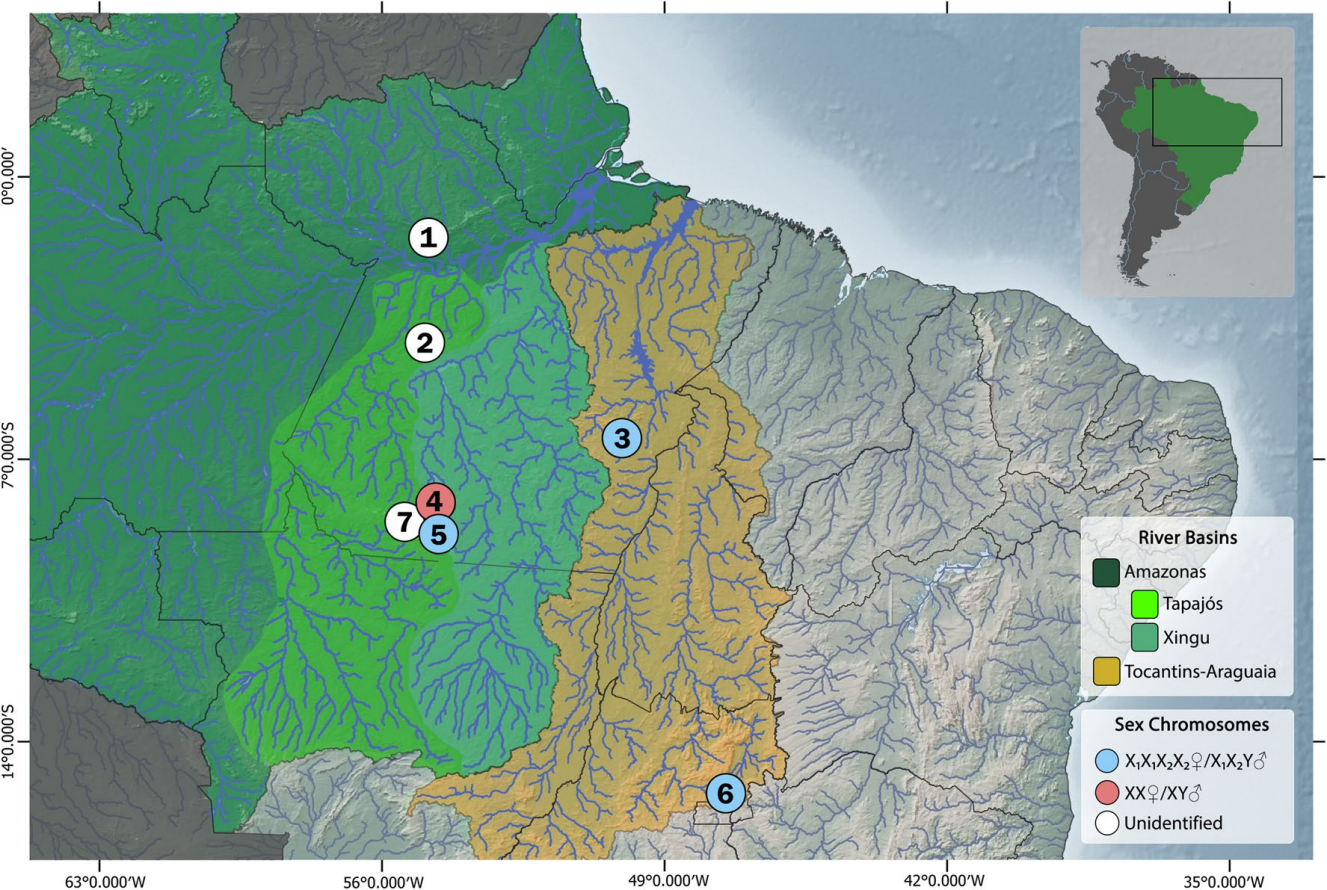
Partial map of Brazil highlighting the Amazonian (green) and Tocantins-Araguaia (orange) river basins. Circles correspond to sampling sites of *Harttia* species whose sex chromosome systems are indicated by specific colors. The color-coding system for river basins and sex chromosomes is presented in frames (bottom right). 1 = *H. guianensis*; 2 = *H. dissidens*; 3 = *H. duriventris*; 4 = *H. rondoni*; 5 = *H. villasboas*; 6 = *H. punctata* and 7 = *Harttia* sp. 3. The map was created with QGIS 3.22 with the package Natural Earth.

Species were gathered from seven distinct localities (Fig. 4, Table 1), with the authorization of the Brazilian environmental agency ICMBIO/SISBIO (License 48628-14) and SISGEN (A96FF09). Mitotic chromosomes were obtained by the classic air-drying method^75^, using the anterior kidney cells as the main source material, being occasionally supplemented with the cells of the spleen tissue. All procedures followed the ethical and anesthesia conducts approved by the Ethics Committee on Animal Experimentation of the Universidade Federal de São Carlos (Process number CEUA 1853260315). The authors complied with ARRIVE guidelines. Specimens were fixed in 10% formalin and deposited in the fish collections of the Instituto Nacional de Pesquisa da Amazônia (INPA-ICT) and Museu de Zoologia da Universidade de São Paulo (MZUSP). Their voucher numbers are provided in Table 1.

**Table 1:** Geographic coordinates, diploid chromosome numbers (2n), karyotype composition, sample sizes (**N**), and voucher numbers of the sampled species.

Species	Geographic Coordinates	2n	Karyotype composition	N	Voucher
2—*Harttia dissidens* **Clade II**	4° 5′ 37.8″ S 55° 0′ 30.2″ W	54♀♂	20 m + 26sm + 8a	07♀25♂	INPA-ICT 059577
*3—H. duriventris* **Clade II**	6° 30′ 06.5″ S 50° 02′ 35.5″ W	56♀55♂	16 m + 16sm + 16st + 8a♀ 17 m + 16sm + 16st + 6a♂	08♀07♂	MZUSP 126598
1—*H. guianensis* **Clade I**	1° 29′ 02.2″ S 54° 50′ 31.2″ W	58♀♂	20 m + 26sm + 2st + 10a	06♀10♂	INPA-ICT 059584
6—*H. punctata* **Clade II**	15° 19′ 25″ S 47° 25′ 26″ W	58♀57♂	16 m + 16sm + 16st + 8a♀ 17 m + 16sm + 16st + 6a♂	10♀12♂	MZUSP 111385
5—*H. villasboas* **Clade II**	8° 44′ 09″ S 54° 57′ 46″ W	56♀55♂	18 m + 24sm + 6st + 8a♀ 19 m + 24sm + 6st + 6a♂	34♀38♂	MZUSP 126599
4—*H. rondoni* **Clade II**	8° 38′ 53″ S 55° 01′ 41″ W	54♀♂	20 m + 26sm + 4st + 4a	15♀14♂	MZUSP 127606
7—*Harttia* sp. 3	08° 39′ 20.7″ S 55° 09′ 24.1″ W	54♀♂	16 m + 18sm + 14st + 6a	11♀15♂	MZUSP 127605

*Harttia* sp. 3 is still waiting for a proper taxonomic identification. Numbers that precede the species names correspond to those in Fig. 1. The species were assigned to their phylogenetic clades following^37^.

### Probe preparation for Zoo-FISH and rDNA FISH

Sex chromosomes of *H. punctata* were selected to be used as probes since this species exhibits the closest 2n relative to the proposed ancestral state for the genus and, at the same time, it possesses a multiple sex chromosome system of the X_1_X_1_X_2_X_2_/X_1_X_2_Y type^28,32,34,35,42^. Fifteen copies of the X_1_ and X_2_ chromosomes were isolated by glass-needle-based microdissection under an inverted microscope (Zeiss Axiovert 135). The collected DNA material was then amplified in a primary degenerated oligonucleotide-primed polymerase chain reaction (DOP-PCR)^76^. The probes were then labeled in the secondary DOP-PCR reaction using 1 µL of the initial amplified product as a template DNA^77^. The probe derived from the X_1_ chromosome (HPU-X_1_) was labeled with Spectrum Orange-dUTP (red), and the one derived from the X_2_ chromosome (HPU-X_2_) with Spectrum Green-dUTP (green) (Vysis, Downers Grove, United States).

The 5S and 18S rDNA fragments were obtained by PCR from the wolf fish *Hoplias malabaricus* genome using primers and thermal profiles described in previous studies^78–80^. The labelling was done by nick translation using Atto550-dUTP (red) for the 5S rDNA and Alexa Fluor 488-dUTP (green) for the 18S rDNA (both Jena Biosciences, Jena, Germany), according to manufacturer's protocol.

### FISH experiments

Zoo-FISH followed the protocol described by Sassi et al.^81^. C_*0*_t-1 DNA prepared from *H. punctata* male genome was used as a blocker to high-copy repeat sequences^82^. Slides with metaphase chromosomes of *H. dissidens*, *H. duriventris*, *H. guianensis*, *H. villasboas*, *H. rondoni* and *Harttia* sp. 3 were denatured in 70% formamide/2 × SSC at 72 °C for 3 min. For each assay, the hybridization solution (200 ng of HPU-X_1_, 200 ng of HPU-X_2_ and 20 µg of C_0_t-1 DNA in 50% formamide + 2 × SSC + 10% dextran sulfate; final volume 20 µL) was denatured for 10 min at 85 °C, cooled at 4 °C for 2 min, and allowed to pre-anneal for 45 min at 37 °C in a thermocycler. Next, the probes were spotted onto the denatured slides, and the hybridization process took place in a dark moist chamber for 48 h at 37 °C. To remove unspecific hybridization signals, slides were washed twice with 1 × SSC at 65 °C (5 min each), and then in 4 × SSC/Tween (5 min) and 1 × PBS (1 min), at room temperature. After capturing the resulting images, the slides were washed for the second round of hybridization^80^, in which 100 ng of each 5S and 18S rDNA probe was applied after being dissolved in the hybridization solution (50% formamide and 10% dextran sulfate in 2 × SSC), in the final volume 20 µL. The rDNA FISH experiments followed the same protocol as described above, except for the hybridization time which was 24 h. In all experiments, chromosomes were finally counterstained with VECTASHIELD^®^ Antifade Mounting Medium with DAPI (4′,6-diamidino-2-phenylindole) (Vector Laboratories, California, United States).

### Microscopy and image analysis

Hybridization patterns were verified in at least 30 metaphase spreads per experiment. Images were captured using an Olympus BX50 microscope (Olympus Corporation, Ishikawa, Japan), coupled with a CoolSNAP camera. Images were processed with Ikaros/ISIS (MetaSystems, Germany). The assignment of signals to specific chromosome pairs, as well as the arrangement of chromosomes into a karyotype, was performed based on data in our previous studies^28,35^.

### Ethical approval

Sample was approved by the Brazilian Environmental Agency ICMBIO/SISBIO (License 48628-14) and SISGEN (A96FF09). All experiments followed the guidelines and were approved by the Ethics Committee on Animal Experimentation of the Universidade Federal de São Carlos (Process number CEUA 1853260315 and 7994170423).

## Data Availability

The datasets generated during and/or analysed during the current study are available from the corresponding author on reasonable request.
